# Diversity of the chiropractic profession in Canada: a cross-sectional survey of Canadian Chiropractic Association members

**DOI:** 10.1186/s12998-022-00463-z

**Published:** 2022-12-09

**Authors:** Danielle Southerst, Nora Bakaa, Pierre Côté, Luciana Macedo, Lisa Carlesso, Joy MacDermid, Silvano Mior

**Affiliations:** 1 Institute for Disability and Rehabilitation Research, Ontario Tech University, Oshawa, Canada; 2 School of Rehabilitation Sciences, McMaster University, Hamilton, Canada; 3 School of Physical Therapy, Western University, London, Canada; 4 Division of Research, Canadian Memorial Chiropractic College, 6100 Leslie Street, Toronto, ON M2H 3J1 Canada

**Keywords:** Cultural diversity, Health equity, Chiropractic, Rehabilitation, Health occupations, Cultural competence

## Abstract

**Background:**

Little is known about the diversity of the chiropractic profession with respect to gender, sexual orientation, race, ethnicity and community of practice. This knowledge is important as profession representation of key equity seeking groups may impact quality of care and access for vulnerable communities. The aim of this cross-sectional survey was to explore the diversity of the chiropractic profession in Canada.

**Methods:**

All registered members of the Canadian Chiropractic Association (*N* = 7721) were invited to participate in a web-based survey between May and June 2021. Survey questions explored diversity with respect to personal demographics (age, sex, gender, sexual orientation, race, ethnicity, language) and practice characteristics (community setting, practice type). Where possible, survey data was compared to data from the 2016 Census of the Canadian population.

**Results:**

We received a total of 3143 survey responses (response rate—41%). The average age of our sample was 44.7 years (standard deviation 12.7). Forty-five percent were female with the same proportion (45.2%) self-identifying as women. Ninety-one percent of the sample self-identified as heterosexual. With respect to race, 80% of respondents were Caucasian. Seventy percent of chiropractors in our sample identified with Canadian ancestry and 29% with European ancestry. In comparison to the Canadian population, some visible minorities were underrepresented. The greatest discrepancy between the Canadian population and our sample was in the proportion of Black and Indigenous chiropractors. With respect to ethnicity, chiropractors identifying themselves with Canadian ancestry were overrepresented in our sample compared to others, specifically those with North American Indigenous and South, Central and Latin American ancestry. Sixty-one percent of chiropractors practiced in major cities and most work in interdisciplinary clinics (42% Complementary and Alternative Medicine and 33% rehabilitation).

**Conclusions:**

This study provides an initial description of diversity within the chiropractic profession in Canada. Women represent less than 50% of the profession nationally. Overall, there is little racial and ethnic diversity in the chiropractic profession compared to the Canadian population, with Black and Indigenous peoples being underrepresented. Future work should focus strategies to foster the development of a more diverse chiropractic workforce.

**Supplementary Information:**

The online version contains supplementary material available at 10.1186/s12998-022-00463-z.

## Background

With an increasingly diverse population in Canada [1], the Canadian Chiropractic Association (CCA) has prioritized the need to develop and achieve cultural competence and address issues of diversity, equity, and inclusion (DEI). In their statement on DEI, the CCA acknowledges cultural competence as a key component in addressing DEI within the profession, and a foundation for effective and equitable delivery of chiropractic care [2]. Cultural competence, at the provider level, involves the demonstration of knowledge, attitudes and behaviours based on diverse and relevant cultural experiences [3]. Cultural diversity and experience are primary elements of cultural competence [4]. A healthcare provider’s background and exposure to diverse communities affect their ability to interact with patients in a cross-cultural manner [4, 5]. Therefore, cultural diversity and experience form a foundation for culturally competent care delivery.

Cultural diversity encompasses differences and similarities both between and within groups with respect to race, ethnicity, language, religion, age, gender, sexual orientation, socioeconomic status, and education. Despite rapidly increasing diversity in the general population, there is no available data on the diversity of Canadian chiropractors and their patients. Data from the United States suggests that the chiropractic profession has yet to achieve a level of diversity that reflects the overall population [6]. Differences between chiropractors and the general population with respect to representation of key equity seeking groups (i.e. Black and Indigenous People of Colour, immigrants, gender and sexual minorities and people living with disabilities) may suggest the potential for systemic unjust and avoidable cultural exclusion (inequity) during the process of becoming a chiropractor [7, 8]. Moreover, a lack of representation of key equity-seeking groups amongst practicing chiropractors could impact quality of care and access to services for vulnerable communities [9–15].

Exploring the diversity of Canadian chiropractors is necessary to provide a preliminary understanding of cultural competency within the profession. Therefore, the aim of this cross-sectional survey is to explore the diversity of the chiropractic profession in Canada. In addition, by comparing the diversity of the profession to the Canadian population, we aim to identify discrepancies in representation of key equity seeking groups.

## Methods

### Study design

We used a cross-sectional study design to survey members of the CCA between May 12, 2021 and June 24, 2021. We used the STrengthening of Reporting in OBservational Studies (STROBE) checklist for cross-sectional studies [16] and the CHEcklist for Reporting Results of Internet E-Surveys (CHERRIES) [17] to prepare our report.

### Ethics

Ethics approval was obtained from Canadian Memorial Chiropractic College (Project # 2,102,003), Ontario Tech University (Project # 16,392), and the Hamilton Integrated Research Ethics Board (Project # 13,042).

### Participants

All registered members of the CCA in 2021 were invited to participate. As of January 2021, there were 7721 members, representing approximately 85% of chiropractors licensed to practice in Canada.

### Recruitment

We developed a schedule for recruitment and follow-up reminders based on the modified tailored design method described by Dillman et al. [18]. In collaboration with the CCA, we developed a strategy to engage members ahead of the survey launch using the Association’s social media pages (i.e., Facebook, Instagram), their biannual e-newsletter *BackMatters* and through emails circulated by the CCA. With the support of the CCA, we also engaged provincial associations. The CCA also enlisted the support of key influential members who have been noted to have a strong social media presence within the profession to emphasize the importance of participation on their social media pages. Survey links were provided to members via email and social media three weeks after the launch of the awareness campaign. Survey links were provided again at 4, 14, 25, and 41-day intervals following the date of initial invitation. To maximize recruitment, participants had a choice to enter a draw for one of ten one-year paid CCA memberships. To minimize burden on respondents, adaptive questioning was accomplished where possible using skip logic for related questions. To enhance completeness of our data, all demographic questions were mandatory with the option to select ‘prefer not to answer’ if desired. Participants were able to review and change their responses using the ‘Back’ button if necessary.

### Survey instrument

We developed a 57-item questionnaire designed to explore the four constructs of the cultural competence conceptual framework first explained by Schim and Miller in 1999 [19]: namely, (1) cultural diversity experience; (2) cultural awareness; (3) cultural sensitivity; and (4) cultural competence behaviours. We report findings related to cultural diversity experience herein.

For cultural diversity experience, we asked a series of questions related to personal and practice demographics. We used a series of self-identification questions related to sex at birth, gender, sexual orientation, disability, race, ethnicity (i.e., ethnic or cultural origin of one’s ancestors), and spoken language proficiency. Where possible, we adopted standard language and definitions used by Statistics Canada to facilitate comparison of our sample to the Canadian population. All questions were critically reviewed by the Canadian Physiotherapy Association’s Global Health Division and Indigenous Health Sub-Committee, as well as a subject matter expert from Director of the Centre for Hate, Bias and Extremism at Ontario Tech University to ensure wording was inclusive and respectful. Questions pertaining to practice demographics focused on geographic location, years of experience, and type of practice (e.g., interdisciplinary vs. solo practice). We pilot tested our survey using a convenience sample of 16 CCA members from across Canada. Efforts were made to select a sample that was diverse with respect to age, gender, province, and years in practice. Both English and French translations of the survey were tested. Feedback was used to improve clarity and functionality of the survey.

### Survey method

We used a commercially available private online survey platform, LimeSurvey© [20], to develop and administer our web-based survey. The survey was open (i.e., no passwords or verification was required) and the survey URL was accessed using a computer or mobile device via Internet or cellular connection. Participants were first directed to the letter of invitation that provided project information and provided informed consent by continuing with the survey. The survey link was circulated in five rounds between May 24 to June 24, 2021. All survey responses were encrypted and stored on a private institutional server with restricted access.

### Statistical analysis

De-identified data was downloaded to an Excel file and responses were numerically coded. Coded data were uploaded into Stata/BE 17 [21] for analysis. Results were reported as descriptive statistics (percentages, means, standard deviations (SD), and/or distributions) for each of the survey questions. Where appropriate, results are reported nationally and by province. We extracted data from the Statistics Canada 2016 Population Census [22] into tables for race and indigenous identity, ethnicity, and language. Prevalence Ratios (PR) with 95% Confidence Intervals (CI) were calculated to facilitate comparison of Census data with our sample demographics [23]. PR less than 1.0 indicate underrepresentation in the sample population.

## Results

### Survey validation

Given this was an open online survey, we made efforts to remove duplicate responses to account for participants who may have inadvertently completed the survey twice, while retaining separate respondents who may have used the same computer. Duplicate data (*n* = 494) was removed based on a set of a priori rules: *Rule 1*: If the IP address and the Response ID (assigned by the survey platform) were the same, one of the responses was deleted; *Rule 2*: If the IP addresses were the same, and one of the responses was blank, the blank response was deleted; *Rule 3*: If the IP addresses and the demographics (age, sex, gender, and race) were the same, and one of the responses was incomplete, we kept the complete response; and *Rule 4*: If the IP addresses and the demographics were the same, and both responses were complete, then we kept the most recent response.

### Survey respondents

**Fig. 1:**
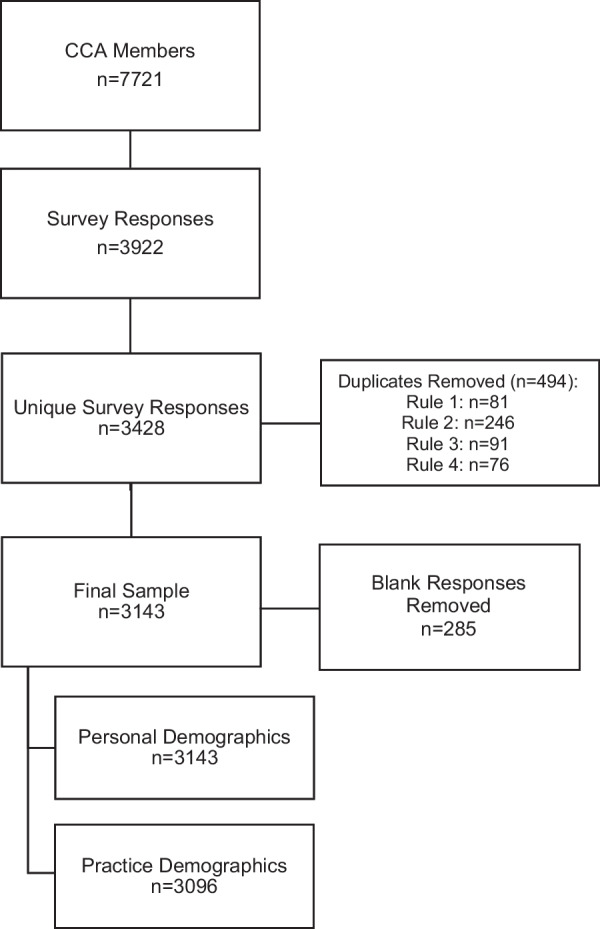
Survey Recruitment

We received a total of 3922 survey responses (Fig. 1). After the removal of duplicates (*n* = 494) and blank responses (*n* = 285), our sample included 3143 participants corresponding to a response rate of 41% of CCA members. All participants completed the section on personal demographics and 99% completed the section on practice demographics. Complete survey responses (including all sections related to cultural competence) were submitted by 77% (*n* = 2432) respondents.

### National distribution

Ontario chiropractors comprised the largest proportion of respondents, followed by Alberta, British Columbia, and Quebec (Table 1). Compared to 2021 CCA membership data, chiropractors from Alberta and British Columbia were overrepresented in our sample, while chiropractors from Ontario and Quebec were underrepresented.

**Table 1:** National distribution of sample compared with CCA membership and the Canadian population

Province	CCA membership; *n* (%)^a^ (*n* = 7764)	Study Sample; *n* (%) (*n* = 3096)	2016 census; *n* (%)^b^ (*n* = 36,991,981)	Prevalence ratio [95% CI]^c^
Alberta	1106 (14.25)	556 (17.96)	4,262,635 (11.52)	1.68 [1.53, 1.84]
British Columbia	1163 (14.98)	556 (17.96)	5,000,879 (13.52)	1.40 [1.28, 1.53]
Manitoba	230 (2.96)	104 (3.36)	1,342,153 (3.63)	0.92 [0.76, 1.12]
New Brunswick	101 (1.30)	38 (1.23)	775,610 (2.10)	0.58 [0.42, 0.80]
Newfoundland and Labrador	68 (0.88)	17 (0.55)	510,550 (1.38)	0.39 [0.24, 0.64]
Nova Scotia	162 (2.09)	67 (2.16)	969,383 (2.62)	0.82 [0.65, 1.05]
Ontario	3636 (46.83)	1380 (44.57)	14,223,942 (38.45)	1.29 [1.20, 1.38]
Prince Edward Island	15 (0.19)	6 (0.19)	154,331 (0.42)	0.46 [0.21, 1.03]
Quebec	1055 (13.59)	281 (9.08)	8,501,833 (22.98)	0.33 [0.30, 0.38]
Saskatchewan	218 (2.81)	84 (2.71)	1,132,505 (3.06)	0.88 [0.71, 1.10]
Northwest Territories, Nunavut and Yukon Territories	6 (0.08)	7 (0.23)	118,160 (0.32)	0.71 [0.34, 1.48]

^a^Based on 2021 membership data supplied by the CCA

^b^Based on Statistics Canada 2016 Census of Population [22]

^c^Prevalence ratio of our sample compared to the 2016 Canadian Census

### Personal demographics

The average age of our sample was 44.7 years (standard deviation (SD) 12.7). Compared to 2021 CCA membership data, chiropractors in the 20–30 and 31–40 age groups were overrepresented in our sample (Table 2). With respect to sex, male chiropractors accounted for just over half of the overall sample; and findings were similar for gender (Table 3). The proportion of chiropractors identifying as women was highest in New Brunswick and lowest in Saskatchewan (data not shown). Very few chiropractors identified as a gender minority; 91% of chiropractors surveyed identify as heterosexual and 6% identified as a sexual minority (i.e., asexual, bisexual, gay, lesbian, pansexual, queer, questioning or unsure).

**Table 2:** Age distribution of sample compared with the CCA membership

Age group	CCA membership; *n* (%)^a^ (*n* = 7764)	Study sample; *n* (%) (*n* = 3103)
20–30	469 (6.0)	459 (14.8)
31–40	1680 (21.6)	843 (27.2)
41–50	2055 (26.5)	844 (27.2)
51–60	1452 (18.7)	532 (17.2)
61–70	792 (10.2)	344 (11.1)
71–80	176 (2.3)	72 (2.3)
> 80	18 (0.2)	6 (0.2)

^a^Based on 2021 membership data supplied by the CCA

**Table 3:** Age, sex, and gender identity of study sample compared with the Canadian population

Demographics	Response category	Sample^a^	2016 Census^b^ (*n* = 35,151,730)	Prevalence ratio [95% CI]^c^
Age	Mean (SD); years	44.7 (12.7)	41.0 (–)^d^	–
Sex	Female; *n* (%)	1400 (45.0)	17,887,530 (50.9)	0.85 [0.79, 0.91]
	Male; *n* (%)	1710 (55.0)	17,264,200 (49.1)	1.18 [1.10, 1.27]
Gender	Woman; *n* (%)	1401 (45.2)	–^d^	–
	Man; *n* (%)	1693 (54.6)	–	–
	Gender Minority; *n* (%)^e^	6 (0.2)	–	–

*SD* Standard Deviation

^a^Study sample sizes: Age: *n* = 3100; Sex: *n* = 3100; Gender (*n* = 3141)

^b^Based on Statistics Canada 2016 Census of Population[22]

^c^Prevalence ratio of our sample compared to the 2016 Canadian Census

^d^No comparable data available in 2016 Census of Population

^e^Gender minority categories collapsed due to small sample sizes: (1) trans-man; (2) trans-woman; (3) gender fluid or non-binary; (4) Indigenous or other cultural gender minority (e.g. Two-spirit); (5) identity not listed

Most chiropractors (77%) identified their race as Caucasian followed by South Asian (5%), Chinese (4%), and mixed race (4%), with less than 1% identifying as Indigenous (Table 4). The proportion of responding non-Caucasian chiropractors was lowest in Quebec (5%) and highest in British Columbia (25%) (Additional file 1: Table S1). Furthermore, the proportion of responding non-Caucasian chiropractors was highest in the 30–39 and 40–49 age groups (24.5% and 23.4% respectively; Additional file 1: Table S2). The majority (80%) of chiropractors identified with Canadian, followed by European (29%), and Asian (14%) ancestry (Table 5). A Euro-Canadian ethnic majority was consistent across the provinces (data not shown).

**Table 4:** Racial distribution of study sample compared with the Canadian population

Race	Study sample; *n* (%) (*n* = 3143)	2016 Census; *n*, (%)^a^ (*n* = 34,460,060)	Prevalence ratio [95% CI]^b^
Indigenous	5 (0.16)^c^	1,673,785 (4.86)^c^	0.03 [0.01, 0.08]
Black	16 (0.52)	1,178,540 (3.48)	0.15 [0.09, 0.24]
Filipino	14 (0.46)	780,125 (2.26)	0.20 [0.12, 0.33]
Latin	10 (0.33)	447,325 (1.30)	0.25 [0.13, 0.46]
South East Asian	19 (0.62)	313,260 (0.91)	0.68 [0.43, 1.06]
Arab	33 (1.07)	523,235 (1.52)	0.70 [0.50, 0.99]
Chinese	122 (3.97)	1,577,060 (4.58)	0.86 [0.72, 1.03]
South Asian	157 (5.11)	1,924,635 (5.59)	0.91 [0.78, 1.07]
West Asian	31 (1.01)	264,305 (0.77)	1.32 [0.93, 1.88]
Korean	25 (0.81)	188,710 (0.55)	1.50 [1.00, 2.21]
Japanese	16 (0.52)	92,920 (0.27)	1.94 [1.19, 3.17]
Caucasian	2467 (80.33)	26,785,480 (77.73)	1.17 [1.07, 1.28]
Mixed	125 (4.07)^d^	232,375 (0.67)^e^	6.25 [5.22, 7.47]
Other	36 (1.17)	132,090 (0.38)	3.08 [2.22, 4.28]

^a^Based on Statistics Canada 2016 Census of Population[22]

^b^Prevalence ratio of our sample compared to the 2016 Canadian Census

^c^Sample size for questions on Indigenous status: Study sample: *n* = 3138; 2016 Census: *n* = 34,460,065

^d^Mixed race was defined in the 2016 Census as ‘multiple visible minorities’

^e^Mixed race was defined in our survey as ‘more than one race’ (including non-visible minority races)

**Table 5:** Ethnic distribution of study sample compared with the Canadian population

Ethnicity	Study sample; *n* (%) (*n* = 3143)	2016 Census; *n* (%)^a^ (*n* = 34,460,065)	Prevalence ratio [95% CI]^b^
African	46 (1.46)	1,067,930 (3.10)	0.46 [0.35, 0.62]
Central or West	7 (0.22)	230,110 (0.67)	0.33 [0.16, 0.70]
North	19 (0.60)	355,045 (1.03)	0.58 [0.37, 0.92]
South or East	20 (0.67)	260,145 (0.75)	0.84 [0.54, 1.31]
American	45 (1.43)	377,405 (1.10)	1.31 [0.98, 1.76]
Asian	425 (13.53)	6,095,235 (17.67)	0.73 [0.66, 0.81]
West, Central or Middle Eastern	71 (2.26)	1,011,145 (2.93)	0.77 [0.60, 0.97]
South	135 (4.30)	1,963,330 (5.70)	0.74 [0.63, 0.88]
East or South East	220 (7.00)	3,163,360 (9.18)	0.75 [0.65, 0.85]
Canadian	2203 (70.14)	11,135,965 (32.32)	4.91 [4.56, 5.31]
Caribbean	29 (0.92)	749,155 (2.17)	0.42 [0.29, 0.60]
European	903 (28.75)	19,683,320 (57.12)	0.30 [0.28, 0.33]
British Isles	428 (13.63)	11,211,850 (32.54)	0.33 [0.30, 0.36]
French	66 (2.10)	4,680,820 (13.58)	0.14 [0.11, 0.17]
Western	214 (6.81)	4,600,855 (13.35)	0.47 [0.41, 0.55]
Northern	86 (2.74)	1,201,320 (3.49)	0.78 [0.63, 0.97]
Eastern	233 (7.42)	3,431,245 (9.96)	0.72 [0.63, 0.83]
Southern	175 (5.57)	3,012,375 (8.74)	0.62 [0.53, 0.72]
Indigenous	34 (1.08)	2,130,520 (6.18)	0.17 [0.12, 0.23]
Latin, Central and South American	13 (0.41)	674,640 (1.96)	0.21 [0.12, 0.36]
Oceana	12 (0.38)	85,470 (0.25)	1.54 [0.87, 2.71]
Other	15 (0.5)	N/A	N/A

^a^Based on Statistics Canada 2016 Census of Population[22]

^b^Relative risk ratio of our sample compared to the 2016 Canadian Census

Approximately 36% of respondents reported speaking more than one language, with 21% being bilingual in French and English. Bilingualism was highest in Quebec (93%), New Brunswick (42%) and Nova Scotia (21%) (data not shown). Other commonly spoken languages were Spanish (3%), Canadian Indigenous Languages (3%), Italian (3%), and Cantonese (2%) (Table 6). Forty-three percent of chiropractors who speak more than one language (20% of our overall sample) use those languages to communicate with their patients.

**Table 6:** Languages spoken by study sample compared with canadian population

Language	Study sample; *n* (%)^c^ (*n* = 3141)	2016 Census; *n* (%)^a,b^ (*n* = 34,460,065)	Prevalence ratio [95% CI]^d^
Multilingual	2023 (35.6)	–	–
Bilingual French/English	646 (20.57)	6,216,065 (18.04)	1.18 [1.08, 1.28]
Canadian Indigenous Languages	105 (3.34)	263,845 (0.77)	4.48 [3.69, 5.44]
Punjabi	61 (1.94)	668,240 (1.94)	1.00 [0.78, 1.29]
Farsi	30 (0.96)	252,320 (0.73)	1.31 [0.91, 1.87]
Spanish	104 (3.31)	995,255 (2.89)	1.15 [0.95, 1.34]
Italian	79 (2.52)	574,725 (1.67)	1.52 [1.22, 1.90]
German	48 (1.53)	502,730 (1.46)	1.05 [0.79, 1.39]
Cantonese	64 (2.04)	699,125 (2.03)	1.00 [0.78, 1.29]
Tagalog	18 (0.57)	612,735 (1.78)	0.32 [0.20, 0.51]
Arabic	34 (1.08)	629,055 (1.83)	0.59 [0.42, 0.83]
Mandarin	25 (0.80)	814,450 (2.36)	0.33 [0.22, 0.49]
Portuguese	21 (0.67)	295,955 (0.86)	0.78 [0.51, 1.19]
Other	218 (6.9)	–	–

*SD* Standard Deviation

^a^Based on Statistics Canada 2016 Census of Population[22]

^b^Knowledge of official/unofficial language refers to whether the person can conduct a conversation

^c^Participants asked to identify languages that they considered themselves to be proficient in (definition of proficiency not provided)

^d^Relative risk ratio of our sample compared to the 2016 Canadian Census

There were few chiropractors (3%) that reported a disability. Of those who did, the most common was physical (i.e., mobility, flexibility, dexterity, pain) (50%), followed by sensory (i.e. hearing and seeing) (34%), mental health-related (14%) and cognitive (i.e., learning, developmental, memory) (12%).

### Practice demographics

The average number of years in practice was 17.5 (SD 12.3). Most chiropractors practice within a major city, whereas fewer practice in smaller towns/regional cities or in rural or remote areas (Table 7). Regional distribution varied by province, with higher proportions of chiropractors practicing in rural areas or smaller towns in New Brunswick, Nova Scotia, Prince Edward Island, and the Territories. (Table 7). The majority of chiropractors who responded practice in an interdisciplinary setting, most commonly in complementary medicine clinics (42%) (Table 7). Less than 1% of chiropractors surveyed practice in hospital settings in British Columbia, Manitoba, and Ontario (data not shown).

**Table 7:** Practice demographics of study sample

Demographics	Response category	Study sample (*n* = 3098)
Years in Practice	Mean (SD); years	17.5 (12.3)
Community^a^	Rural/Remote; *n* (%)	300 (9.7)
	Town or Smaller Regional City; *n* (%)	909 (29.3)
	Major City; *n* (%)	1889 (61.0)
Practice Type	Solo; *n* (%)	1040 (33.6)
	Interdisciplinary Rehabilitation; *n* (%)	1020 (32.9)
	Interdisciplinary Complementary; *n* (%)	1299 (41.9)
	Interdisciplinary Medical; *n* (%)	199 (6.4)
	Hospital Out; *n* (%)	22 (0.7)
	Hospital In; *n* (%)	–
	N/A; *n* (%)	8 (0.3)
	Other; *n* (%)^b^	66 (2.1)

*SD* Standard Deviation

^a^Rural/remote = population 1000 to 10,000; Town or smaller regional city = population 10,000 to 100,000; Major city (urban/metropolitan/suburban) = population > 100,000

^b^Not currently practicing

### Comparison with the Canadian population

With respect to sex, females were underrepresented in our sample compared to the Canadian population (PR 0.85; 95% CI 0.79, 0.91). Moreover, chiropractors over 60 years of age were underrepresented whereas chiropractors under the age of 50 were overrepresented (Table 8).

**Table 8:** Age distribution of sample compared with the Canadian population

Age Group	Study sample; *n* (%) (*n* = 3100)	2016 Census; *n* (%)^a^ (*n* = 27,286,000)	Prevalence ratio [95% CI]
20–29	348 (11.2)	4,528,680 (16.6)	0.64 [0.57, 0.71]
30–39	881 (28.4)	4,617,760 (16.9)	1.95 [1.80, 2.11]
40–49	795 (25.6)	4,615,100 (16.9)	1.69 [1.56, 1.84]
50–59	617 (19.9)	5,298,315 (19.4)	1.03 [0.94, 1.13]
60–69	362 (11.7)	4,262,990 (15.6)	0.71 [0.64, 0.80]
70–79	91 (2.9)	2,442,725 (9.0)	0.31 [0.25, 0.38]
80 +	6 (0.2)	1,520,430 (5.6)	0.03 [0.01, 0.07]

^a^Based on Statistics Canada 2016 Census of Population using population 20 years and older[22]

A number of racial minorities were significantly underrepresented in our sample when compared to the Canadian population (Table 4). Underrepresentation was most pronounced amongst people who identify themselves as Indigenous (PR 0.03; 95% CI 0.01, 0.08), Black (PR 0.15; 95% CI 0.09, 0.24), Filipino (PR 0.20; 95% CI 0.12, 0.24), and Latin American (PR 0.25; 95% CI 0.13; 0.46).

With respect to ethnic origins, chiropractors with Canadian ancestry were significantly overrepresented (PR 4.91; 95% CI 4.56, 5.31) (Table   5). Conversely, a number of ethnic origins were significantly underrepresented in our sample. Underrepresentation was most pronounced for North American Indigenous (PR 0.17; 95% CI 0.12, 0.23) and Latin, Central and South American (PR 0.21; 95% CI 0.12, 0.36) and European (PR 0.30; 95% CI 0.28, 0.33) ethnic origins.

Of the most commonly spoken languages in Canada, most were adequately represented within our sample (Table 6). However, people who speak Tagalog (PR 0.30; 95% CI 0.20, 0.51), Arabic (PR 0.60; 95% CI 0.42; 0.83), and Mandarin (PR 0.3; 95% CI 0.22, 0.49) were underrepresented in our sample when compared with the Canadian population. Conversely, Canadian Indigenous language speakers were significantly overrepresented in our sample (PR 4.50; 95% CI 3.69, 5.44).

## Discussion

We found most chiropractors in our survey were between 31 and 50 years old. Few identified themselves as a sexual or gender minority, with about half identifying as women and most identifying as heterosexual. Most respondents were Caucasian, and the majority identified their ethnic origins to be either Canadian or European. Compared to the Canadian population, visible minorities were underrepresented in our sample; especially Black and Indigenous chiropractors. Furthermore, people of North American Indigenous, Latin American and European ethnic origins were also underrepresented in our sample. Conversely, chiropractors of Canadian ancestry were overrepresented in our sample. These results may have implications for cultural competence and the equitable delivery of chiropractic care to patients from equity seeking groups.

Our results also have implications for the availability of chiropractic care in rural Canada. Fewer than 10% of our sample reported practicing within a rural or remote region (populations < 10,000). According to the 2016 Census, 18.7% of the population lives in a rural area (population < 1000) [24]. Given the difference in definitions used, our findings may overestimate the number of chiropractors practicing in rural Canada and point to the potential for decreased access to chiropractic care for segments of the population living within rural areas. This is an important finding given that Canadians living in rural regions, especially those in northern communities, experience significant health disparities, yet have less access to primary and specialty health care [25–27]. Past research has highlighted the importance of understanding strategies to enhance the recruitment and retention of health providers to enhance access to care within rural Canada [28].

### Comparison with other samples

In 1993, the United States National Board of Chiropractic Examiners (NBCE) conducted a job analysis of the chiropractic profession in Canada [29]. Surveys were sent to 982 licensed chiropractors from across Canada (excluding the Territories), and 683 responded (69.6% response rate), representing 21% of licensed chiropractors in Canada. The analysis provided limited demographic information about practicing chiropractors, reporting 87% were male [29]. Our findings suggest increased representation of females within the profession. Although the Canadian chiropractic profession has not quite achieved gender parity, data suggest that progress may be slower in the US. A practice analysis from the US indicated that in 2019, the profession continued to be male dominated (67% male) despite a consistent increase in female representation since 1991 [6]. Our findings mirror data from the US related to gender minorities, where very few chiropractors (0.2%) self-identify as transgender or non-binary [6].

The NBCE’s 1993 job analysis found that 88.1% of Canadian chiropractors were born in Canada [29]. In our sample, 70% of respondents identified themselves as having Canadian ancestry. Although it is challenging to compare these figures directly, both studies suggest a lack of ethnic diversity within the profession. We could not identify available data on racial representation within the Canadian chiropractic profession. In 2019, 91% of chiropractors in the US were White; just 1.6% were Black, 3.0% were Hispanic/Latino and 0.9% were Native American [6]. Although our findings suggest greater racial diversity in Canadian compared to US chiropractors, there is similar underrepresentation of Black, Latin, and Indigenous peoples compared to the national population.

Data on diversity within other health professions in Canada is not commonly reported [11, 30, 31]. However, in a recent survey of Canadian physiotherapists, we found a similar lack of representation of visible minorities [32]. Moreover, similar trends have been reported in the medical profession in the United States where underrepresented minorities include people who are Black, Hispanic, and Native American [33].

### Implications

Our findings have implications for professional leadership and future research. Results suggest the need to investigate strategies that could foster a more diverse chiropractic workforce, particularly the underrepresentation of Black and Indigenous peoples. Although a higher proportion of responding non-Caucasians in younger age groups in our sample suggests a higher proportion of visible minorities in more recently recruited chiropractors, we were unable to specifically assess whether representation is improving among Black and Indigenous peoples. In 2015, the Truth and Reconciliation Commission of Canada called upon health professions to increase the recruitment and retention of Indigenous peoples, particularly within Indigenous communities [34]. Our findings support this call to action within the chiropractic profession and its educational institutions. Of note, underrepresentation of indigenous peoples within the chiropractic profession may not only reflect underlying inequity in educational opportunities but also a lack of integration of indigenous practices into community-based chiropractic care [35] and underlying cultural and structural differences. Interestingly, despite the underrepresentation of indigenous chiropractors in our sample, we found the reported use of Canadian Indigenous language to be overrepresented. The explanation for this finding is unclear and therefore requires further investigation. Under-representation of Black people in the Canadian chiropractic profession is also relevant given evidence of systemic discrimination and persistent undertreatment of pain in Black people [36, 37]. Given the evidence that racial concordance improves patient experience and satisfaction in Black people [9, 14], increased diversity in the chiropractic profession could impact access to quality care for painful musculoskeletal conditions.

Although numerous commentaries and narrative reviews emphasize the importance of focused efforts on improving diversity within the profession, little work has been done to identify specific priorities and strategies to accomplish these objectives, particularly within the Canadian context [38–42]. One strategic example can be seen in the medical profession, where Canadian Medical schools have taken the lead, implementing programs targeted at recruitment and retention of racialized minorities, in particular Black and Indigenous students [11, 43]. Should similar strategies be implemented by chiropractic institutions, routinely collected demographic data will allow for ongoing evaluation. While improving diversity may be a lengthy process [11], there may be a need to explore more immediate strategies to foster the development of a culturally congruent model of chiropractic care.

Cultural congruence refers to the dynamic interaction between the cultural competence of providers, the views, experiences and expectations of patients, and the sociocultural environment in which the clinical interaction takes place [5]. Although diversity and cultural experience impact cultural competence, developing cultural awareness and adopting culturally competent behaviours are crucial, and may be amenable to education and training [44, 45]. Furthermore, the diversity and unique cultural experiences of patients contribute greatly to overall cultural congruence of interactions [4, 5]. However, there is currently limited data on the cultural characteristics of patients who utilize chiropractic care in Canada. This information can be used to identify potential gaps in access to chiropractic care as well as informing the cultural needs of people who generally access services. Our study provides a foundation for future work to explore provider and patient-level attributes that may contribute to a model of culturally congruent chiropractic care.

Lastly, to support a culturally congruent model of chiropractic care, we need to ensure that our professional organizations and institutions are culturally competent. Future work may focus on assessing the capabilities and readiness of chiropractic professional organizations and educational institutions to support the advancement of the profession with respect to DEI.

### Limitations

We acknowledge the possibility of response bias in our study. We recruited 41% of the CCA membership. Compared with CCA membership data, chiropractors from British Columbia and Alberta were overrepresented in our sample, whereas chiropractors from Ontario and Quebec were underrepresented. Furthermore, chiropractors in the 20–30 and 31–40 age groups were overrepresented in our sample. Given the differences observed in the proportion of visible minorities by province and across age groups, our findings may overestimate the racial diversity CCA members. Another limitation is that we surveyed only CCA members, who comprise about 85% of all Canadian chiropractors. In consideration of the response rate, it is possible that the diversity of the entire profession may differ. To protect anonymity, we did not verify membership status. To reach members, we circulated the survey link using email and member-only social media sites; however, it is possible that some respondents were not members of the CCA. Further, we allowed multiple responses from one IP address to accommodate chiropractors who completed the survey from an office where multiple chiropractors practice. We developed a systematic approach to the removal of duplicates but acknowledge that duplicate responses may have been retained and, conversely, some independent responses may have been lost in this process. Finally, formal definitions of terms such as ‘race’ and ‘ethnicity’ were not provided in our survey which may have led to misclassification bias.

## Conclusions

Our findings provide an initial description of diversity within the chiropractic profession in Canada. Although women make up nearly half of the profession nationally, very few identify as a gender minority. Overall, the profession lacks racial and ethnic diversity compared to the Canadian population, with Black and Indigenous peoples underrepresented. Findings from this study suggest a need to focus efforts on developing strategies to address diversity within the chiropractic profession in Canada, in particular, improving representation of Black and Indigenous peoples.

## Supplementary Information

**Additional file 1:** Racial Distribution of Canadian Chiropractors.

## Data Availability

The datasets generated from our study sample are not publicly available due to the risk to participants’ confidentiality. Selected data are available from the corresponding author upon reasonable request. The datasets analyzed in the comparison of our sample to the Canadian population are available from Statistics Canada Census Program, Data products, 2016 Census: https://www12.statcan.gc.ca/census-recensement/2016/dp-pd/index-eng.cfm [22].

## Abbreviations

CCA: Canadian Chiropractic Association
DEI: Diversity, equity and inclusion
STROBE: STrengthening of Reporting in OBservational Studies
CHERRIES: CHEcklist for Reporting Results of Internet E-Surveys
SD: Standard deviation
PR: Prevalence ratio
CI: Confidence interval
NBCE: National board of chiropractic examiners
