# Supplementary material for: Diversity of the chiropractic profession in Canada: a cross-sectional survey of Canadian Chiropractic Association members
Source: Chiropr Man Therap. 2022 Dec 9;30:52. doi: 10.1186/s12998-022-00463-z (PMC9733254; doi:10.1186/s12998-022-00463-z)
Supplement: Supplementary file 1 — Additional file 1: Racial Distribution of Canadian Chiropractors. [file 12998_2022_463_MOESM1_ESM.docx]

**Addition file 1: Racial Distribution of Canadian Chiropractors.**

**Table S1. Racial Distribution of Canadian Chiropractors Sampled by Province**

| **Race** | **Sample; n (%)** | **Province; n (%)^1^** | | | | |
| --- | --- | --- | --- | --- | --- | --- |
|  |  | **AB** | **BC** | **MB** | **ON** | **QC** |
| Caucasian | 2467 (80.3) | 431 (79.8) | 410 (74.7) | 84 (82.4) | 1047 (77.8) | 265 (95.0) |
| Non-Caucasian | 604 (19.7) | 125 (20.2) | 139 (25.3) | 18 (17.6) | 298 (22.2) | 14 (5.0) |

Abbreviations: AB – Alberta; BC – British Columbia; MB – Manitoba; ON – Ontario; QC – Quebec

^1^Data no shown for Saskatchewan, Nova Scotia, New Brunswick, Newfoundland and Labrador, Prince Edward Island, Northwest Territories, Yukon and Nunavut due to low number of responses

**Table S2. Race of Canadian Chiropractors Sampled by Age Group**

| **Race** | **Sample; n (%)**  **(n=3071)** | **Age Group; n (%)** | | | | | | |
| --- | --- | --- | --- | --- | --- | --- | --- | --- |
|  |  | **20-29** | **30-39** | **40-49** | **50-59** | **60-69** | **70-79** | **80+** |
| Caucasian | 2467 (80.3) | 276 (79.3) | 675 (76.6) | 600 (75.5) | 481 (78.0) | 320 (88.4) | 74 (81.3) | 6 (100.0) |
| Non-Caucasian | 604 (19.7) | 72 (20.7) | 206 (23.4) | 195 (24.5) | 136 (22.0) | 42 (11.6) | 17 (18.7) | -- |
